# Supplementary material for: Identification of photoperiod-induced specific miRNAs in the adrenal glands of Sunite sheep (Ovis aries)
Source: Front Vet Sci. 2022 Jul 22;9:888207. doi: 10.3389/fvets.2022.888207 (PMC9354845; doi:10.3389/fvets.2022.888207)
Supplement: Supplementary file 1 [file Data_Sheet_1.ZIP › Supplementary file/miRDeep2 file.zip!/miRDeep2 file/Know_Structure/SPLP42A1/oar-mir-431.pdf]

miRBase precursor : oar-mir-431
Total read count : 202
oar-miR-431 read count : 18
remaining reads : 184

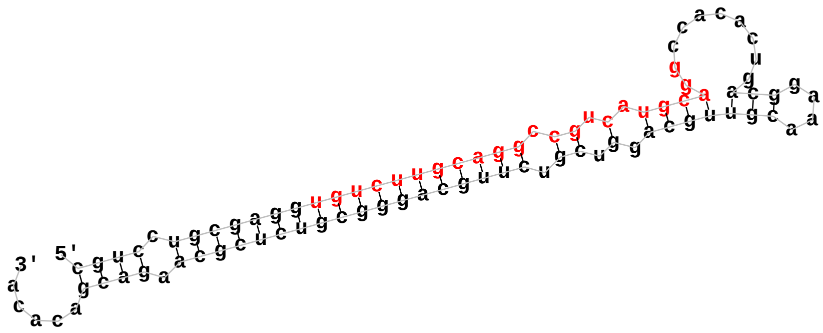

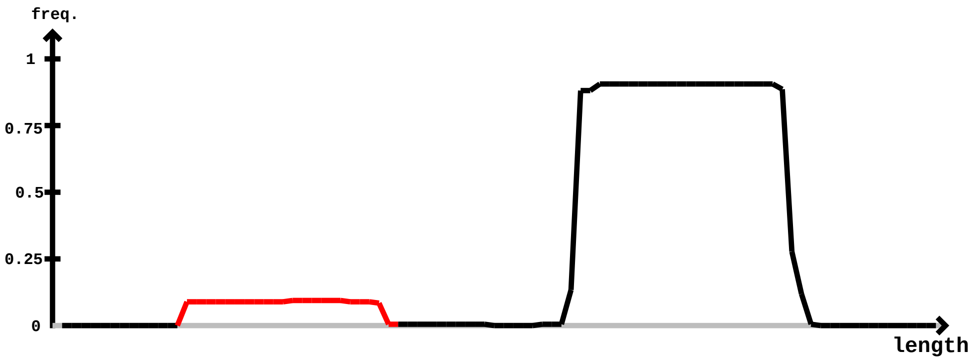


```
                oar-miR-431
5'- cguccugcgaggugucuugcaggccgucaugcaggccacacugacggaaacguugcaggucgucuugcagggcgucucgcaagacgacaca   -3'    exp
    ((((.((((((((((((((((((.((.(.((((..........(((....))))))).).)).)))))))))))))))))).)))).....    reads      mm         sample
    ............ugucuugcaggccguca..............................................................    1          0          seq
    ............ugucuugcaggccgucaugc...........................................................    1          0          seq
    ............ugucuugcaggccgucaugca..........................................................    16         0          seq
    .......................ccgucaugcaggccacacuga...............................................    1          0          seq
    .................................................acguugcaggucgucuugcagggcguc...............    1          0          seq
    ....................................................uugcaggucgucuugcagggcg.................    2          0          seq
    ....................................................uugcaggucgucuugcagggcgu................    20         0          seq
    ....................................................uugcaggucgucuugcagggcguc...............    2          0          seq
    ....................................................uugcaggucgucuugcagggcgucu..............    2          0          seq
    .....................................................ugcaggucgucuugcagggcg.................    2          0          seq
    .....................................................ugcaggucgucuugcagggcgu................    101        0          seq
    .....................................................ugcaggucgucuugcagggcguc...............    27         0          seq
    .....................................................ugcaggucgucuugcagggcgucu..............    20         0          seq
    .....................................................ugcaggucgucuugcagggcgucuc.............    1          0          seq
    .......................................................caggucgucuugcagggcgu................    2          0          seq
    .......................................................caggucgucuugcagggcguc...............    2          0          seq
    .......................................................caggucgucuugcagggcgucu..............    1          0          seq
```
